# Prevalence of and Risk Factors for Age-Related and Anterior Polar Cataracts in a Korean Population

**DOI:** 10.1371/journal.pone.0096461

**Published:** 2014-06-17

**Authors:** Kyung-Sun Na, Yong-Gyu Park, Kyungdo Han, Jee Won Mok, Choun-Ki Joo

**Affiliations:** 1 Department of Ophthalmology and Visual Science, Seoul St. Mary's Hospital, College of Medicine, The Catholic University of Korea, Seoul, Korea; 2 Department of Biostatistics, The Catholic University of Korea, Seoul, Korea; 3 Catholic Institutes of Visual Science, The Catholic University of Korea, Seoul, Korea; 4 Department of Health Promotion Center, Seoul St. Mary's Hospital, College of Medicine, The Catholic University of Korea, Seoul, Korea; Washington University, United States of America

## Abstract

**Objective:**

To investigate the potential risk factors associated with nuclear, cortical, posterior subcapsular, and anterior polar cataracts (APC) in the Korean population.

**Research Design and Methods:**

This was a population-based, cross-sectional study of 7992 adults (over 40 years of age) from the data of the fourth annual Korea National Health and Nutrition Examination Survey, performed from 2007 to 2009. The presence of lens opacity was examined by slit-lamp biomicroscopy and evaluated according to LOCS II standard photographs. The subtype of cataract present, including nuclear, cortical, posterior subcapsular, and anterior polar cataracts, was noted. Multivariable adjusted logistic regression analysis was conducted to examine the odds ratio (OR) and 95% confidence interval (CI) for association of each specific type of cataract with age, sex, health examination, and medical history.

**Results:**

The prevalence of nuclear, cortical, and posterior subcapsular cataract increased gradually with increasing age. However, the prevalence of APC peaked in the 50- to 59-year-old subjects. All types of cataract except for APCs were more prevalent in women. Oral steroid use was associated with a lower risk of APC.

**Conclusions:**

These findings showed the unique characteristics of APC in the Korean population.

## Introduction

Cataracts are the principal cause of blindness and visual impairment worldwide, making it necessary from the public health standpoint to identify risk factors for their development and progression [1]–[3]. Studies on the prevalence of and risk factors for cataracts have been conducted mainly in white European-derived populations in the United States, Australia, and Europe [4]–[11]. Less is known about populations of African, Indian, or East Asian descent [3], [12]–[15].

**Figure 1:**
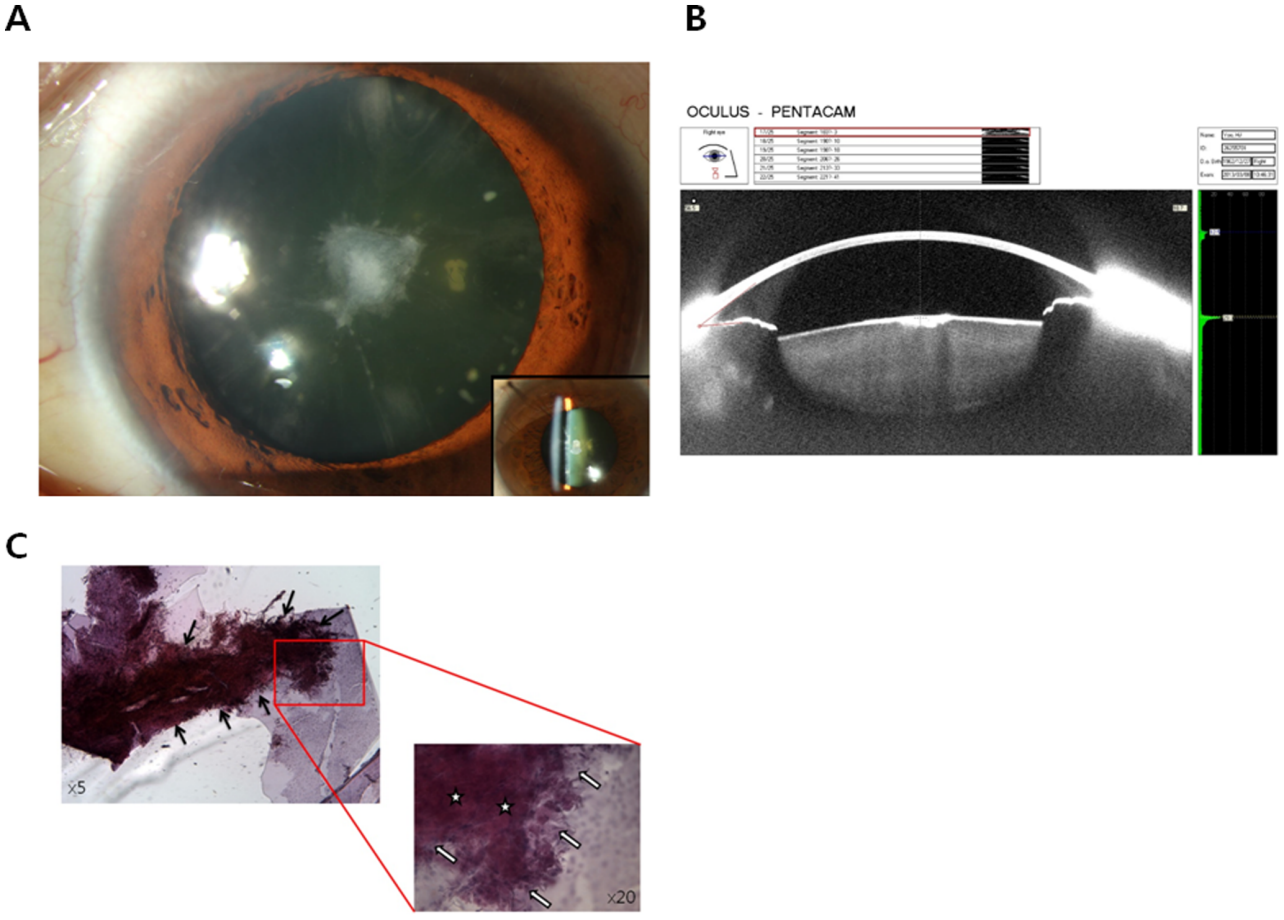
Fifty-year-old man showed findings of anterior polar cataract. (A) Slit-lamp examination demonstrates the form of white-grey opacity appearing under the central part of the anterior lens capsule. (B) Sheimpflug-measured lens density showing focal anterior polar opacity. (C) Light microscopy of the anterior capsule following cataract surgery of anterior polar cataract. Multilamellar arrangement and isolated aggregations of collagenous materials are present (arrows). High-power image shows proliferative changes of the lens epithelial cells (open arrows) and increased collagenous aggregations (stars).

Anterior polar cataract generally refers to the congenital type of capsulolenticular cataract seen in Western countries and has been reported to be related to microphthalmia, aniridia, and bilateral retinoblastoma [2]. However, there is a unique type of cataract with focal opacity at the anterior capsulolenticular area that develops in early adulthood in otherwise healthy Korean patients (Fig. 1(A), (B), and (C)). APC is not well documented even in other Asian countries and is considered unique to Koreans. Previously, we conducted a hospital-based study showing that the prevalence of APC in our patients was 6.02%, and male predominated [16]. Until very recently, Korea was largely considered to be a homogenous, racially intolerant country with little or no experience with large-scale immigration. We have previously shown that the cataractous lens epithelial cells (LECs) of anterior subcapsular cataracts are transdifferentiated into spindle-shaped fibroblast-like cells without cellular junctions and are embedded within a fibrillar meshwork mass [17]. Lens epithelial cells in the anterior subcapsular area fail to migrate to the equatorial area, resulting in the accumulation of several layers of proliferating cells in the anterior subcapsular area. This change is different from the changes in lens fiber proteins in other types of cataracts.

In this study, we aimed to elucidate the prevalence of and risk factors for non-age related anterior polar cataracts in the absence of any general abnormality and those of age-related cataracts, including nuclear and cortical cataracts.

## Materials and Methods

### Study Subjects

This cross-sectional study included a representative sample of the data gathered in the fourth Korea National Health and Nutrition Examination Survey (KNHANES IV), which was performed from 2007 to 2009 by the Division of Chronic Disease Surveillance under the Korea Centers for Disease Control and Prevention.

The sampling units were households selected through a stratified, multistage, probability sampling, based on geographic area, sex, and age group, of household registries. Information was collected from stratified multistage probability samples of Korean households representing the noninstitutionalized civilian population. The survey was composed of 3 parts, that is, a health interview survey, a health examination survey, and a nutrition survey, and was a nationwide representative study of noninstitutionalized civilians, involving a stratified, multistage, probability-sampling design with a rolling survey sampling model. Sampling units were defined based on household unit data from the 2005 National Census Registry, including those for geographic area, sex, and age.

Among 25250 individuals who completed all surveys, total 19386 (76.8%) had an ophthalmic examination. Of the 8801 subjects, who were older than 40 years old, 416 were bilaterally pseudophakic or aphakic; and 393 had mixed-type cataracts. The remaining 5242 free of cataract and 2750 with cataract were included in this study. Institutional Review Board/Ethics Committee approvals for the study were obtained from the Catholic University of Korea in accordance with the Declaration of Helsinki. The data are publicly available from the Korean Centers for Disease Control and Prevention [18].

### Procedures

Questionnaires were used to collect demographic information, smoking history, educational status, weight change after the age of 20 years (gain or loss), daily sunlight exposure, alcohol consumption, sleep duration, stress level, medical history, and any use of prescription drugs or nonprescription medication. Smoking behavior was categorized as current smoker, ex-smoker, or nonsmoker. Alcohol-consumption behavior was classified as nondrinker, mild-to-moderate drinker (1.0–30.0 g alcohol/day), or heavy drinker (>30.0 g alcohol/day) after conversion of the average frequency and amount of alcoholic beverages consumed into the amount of pre-alcohol (in grams) consumed per day. Psychological stress level was investigated according to the subjective cognitive complaints by the questionnaires.

Waist circumference was measured to the nearest 0.1 cm in a horizontal plane at the level of the midpoint between the iliac crest and the costal margin at the end of a normal expiration. The body mass index (BMI) was calculated as the individual's weight in kilograms divided by the square of the individual's height in meters. The BMI was grouped into 2 categories, that is, normal (<25.0 kg/m^2^) and overweight (≥25.0 kg/m).

Systemic hypertension was defined as a measured systolic blood pressure >160 mm Hg and/or a diastolic blood pressure >90 mm Hg or current use of systemic antihypertensive drugs. Diabetes was defined as a measured fasting blood sugar level of >126 mg/dL or current use of antidiabetic medication. Peripheral blood was obtained after fasting for a minimum of 8 hours.

### Definition of Cataract

Lens opacity was diagnosed by trained ophthalmologists by using the Lens Opacity Classification System (LOCS) II system [19]. The Lens Opacities Classification System II (LOCS II) was used to classify opacities until into 7 cortical (C0, Ctr, CI, CII, CIII, CIV, CV), 5 nuclear (NO, NI, NII, NIII, NIV), and 5 PSC (P0, PI, PII, PIII, PIV) grades of increasing severity, according to photographic standards. Aphakia or pseudophakia were also documented even though excluded in the current study. The pupils were dilated with 1% tropicamide/2.5% phenylephrine hydrochloride eye drops, and the presence of lens opacity was examined by slit-lamp biomicroscopy and evaluated according to LOCS II standard photographs. The severity or grade of lens opacity was not recorded, and only the subtype of cataract present, including nuclear, cortical, posterior subcapsular, and anterior polar cataracts, was noted.

The definitions of lens opacities used in this study were similar to those used in the previous study [20], [21]. Nuclear, cortical, posterior subcapsular cataract (PSC), and APC cataracts were recorded in individuals with the same single type of opacity present in both eyes. They were defined in individuals with only a single type opacity with a LOCS II≥2, presented between both eyes. If a participant had unilateral lens extraction, the contralateral phakic eye was used to define the lens opacity type in that individual. Subjects with mixed-type opacities or bilateral lens extraction were excluded.

### Histological Examination of Anterior Capsule

The extracted anterior capsules were fixed for 10 min in 4% formaldehyde in PBS and permeabilized in PBS containing 0.5% Triton X-100, also for 10 min. The specimens were stained with hematoxilin and eosin. Images were captured and analyzed with light microscope.

### Statistical Analysis

The data are expressed as numbers and percentages (categorical) or the mean ± standard error (continuous). Multivariable adjusted logistic regression analysis was conducted to examine the odds ratio (OR) and 95% confidence interval (CI) for association of each specific type of cataract with age, sex, smoking history, education, weight change, sunlight exposure, alcohol consumption, sleep duration, stress level, BMI, waist circumference, estrogen use (women only), NSAID use, other anti-inflammatory medication use, vitamin use, hypertension, and diabetes. Age and sex were used as covariates for calculating the adjusted OR.

Because the KNHANES III included weights to compensate for the complex sampling design and to allow approximations of the Korean population, weighted analyses were performed with the SAS software. All statistical analyses were performed with the SAS (version 9.2; SAS Institute, Cary, NC, USA) survey procedure to account for the complex sampling design and provide nationally representative prevalence estimates. A P value<0.05 was considered statistically significant.

## Results

### Baseline Characteristics

The clinical characteristics of the study participants are presented in Table 1. The numbers of participants analyzed were 1783, 807, 56, and 104 for subjects with nuclear cataracts, cortical cataracts, PSCs, and APCs, respectively.

**Table 1:** Baseline characteristics of study participants.

	Nuclear (n = 1783)	Cortical (n = 807)	PSC (n = 56)	APC (n = 104)
**Age**				
40–49	13.8(0.6)	15.5(0.6)	16.5(0.6)	16.6(0.6)
50–59	14.9(0.6)	17.6(0.6)	18.7(0.6)	28.4(0.7)
60–69	28.7(0.8)	28.6(0.8)	28.5(0.7)	18.5(0.6)
≥70	42.6(1.2)	38.2(1.1)	36.3(1.1)	36.5(1.1)
**Sex**				
Male	48.2(0.7)	48.0(0.6)	47.9(0.6)	52.2(0.6)
**Smoke**				
Never	77.4(0.7)	77.3(0.6)	77.6(0.6)	77.6(0.6)
Current	22.6(0.7)	22.7(0.6)	22.4(0.6)	22.4(0.6)
**Education**				
High school to college graduate	31.6(1.9)	27.9(2.8)	39.9(10.1)	37.4(7.0)
**Weight change** †				
Gain>10 kg	0.5(0.2)	0.4(0.4)	.	.
Gain≤10 kg	9.9(0.9)	11.7(1.5)	11.1(5.3)	10.1(3.2)
No change	72.7(1.3)	71.7(2.1)	68.5(7.1)	66.8(5.8)
Loss≤10 kg	16.3(1.1)	15.6(1.6)	20.5(6.4)	23.1(4.5)
Loss >10 kg	0.5(0.1)	0.6(0.3)	.	.
**Daily sunlight exposure**				
More than 6 hours	35.5(2.1)	41.5(3.8)	32.5(8.4)	48.2(8.7)
**Alcohol** ‡				
Never drinker	40.5(1.6)	39.6(2.2)	32.4(8.1)	44.9(4.5)
Mild to Moderate drinker	50.3(1.6)	50.5(2.4)	65.6(8.6)	44.9(4.9)
Heavy drinker	9.2(1.1)	9.8(1.4)	2.0(1.5)	10.1(3.3)
**Daily sleep**				
4 hours or less	3.2(0.5)	2.5(0.6)	4.7(3.0)	0.6(0.6)
4–6 hours	20.8(1.2)	18.1(1.6)	24.1(7.3)	17.0(4.1)
More than 6 hours	75.9(1.3)	79.4(1.6)	71.2(7.7)	82.4(4.1)
**Stress level**				
Excessive	6.3(0.7)	6.1(1.0)	4.0(4.0)	7.3(2.8)
Much	20.5(1.1)	20.5(1.7)	30.2(7.8)	12.6(3.7)
Frequent	50.3(1.6)	51.6(2.1)	40.3(8.6)	62.3(5.7)
Rare to never	23.0(1.3)	21.7(1.9)	25.5(7.3)	17.8(4)
**BMI**				
≥25 kg/m^2^	34.3(2.1)	36.3(1.4)	45.2(5.3)	34.6(8.3)
**Waist circumference**				
≥90 cm in male, ≥80 cm in female	40.0(2.5)	48.4(1.6)	45.9(5.9)	43.1(8.4)
**Estrogen use (women only)**				
Yes	12.6(1.4)	12.7(1.9)	11.1(7.1)	6.9(3.0)
**Steroid use**				
Yes	77.4(2.2)	80.3(3.1)	90.5(6.7)	56.3(10.6)
**NSAIDs use**				
Yes	66.3(4.7)	63.3(7.7)	59.6(27.4)	54.7(14.5)
**Hypertension**				
Yes	38.1(1.6)	36.4(2.0)	31.9(7.4)	38.1(5.6)
**Diabetes**				
Yes	15.7(1)	12.7(1.4)	26.7(7.5)	9.4(2.9)
**Vitamin use**				
Yes	80.4(1.3)	81.9(2.2)	84.9(8.7)	70.6(5.8)

Data are expressed as percentage (SE).

PSC = posterior subcapsular opacity; APC = anterior polar cataract; BMI = body mass index; NSAIDs = nonsteroidal anti-inflammatory drugs.

† Weight change after the age of 20 years.

‡ Classified as nondrinker, mild-to-moderate drinker (1.0–30.0 grams alcohol/day), or heavy drinker (>30.0 grams alcohol/day) after conversion of the average frequency and amount of alcoholic beverages consumed into the amount of pre-alcohol (in grams) consumed per day.

**Figure 2:**
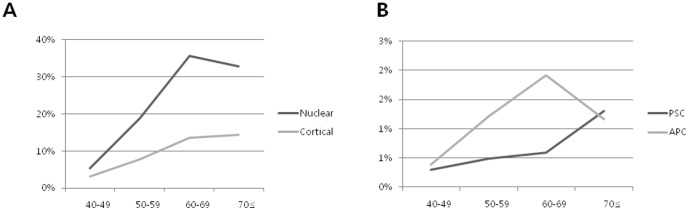
Linear graphs showing age distribution of cataract. (A) Age distribution of nuclear cataract and cortical cataract in Korean population. Both types of cataract show a highest incidence at the age of 60–69. (B) Age distribution of posterior subcapsular cataract (PSC) and anterior polar cataract (APC). PSC shows a gradual increase according to age, and APC present a highest peak at the age of 50–59. * PSC = posterior subcapsular opacity; APC = anterior polar cataract.

The prevalence of age-related cataracts, including nuclear cataract, cortical cataract, and PSC increased gradually with increasing age. However, the prevalence of APC peaked in the 50- to 59-year-old subjects.(Fig. 2(A),(B)) All types of cataract except for APCs were more prevalent in women; the incidence of APC was greater in men than in women (1.12% vs. 0.96%). The incidence of APC was relatively lower in subjects using estrogen (woman only) or NSAIDs. Diabetes was more frequent in subjects with PSCs than in those with other types of cataracts.

### Significant Associations with Cataracts

Table 2 demonstrates the results of logistic regression models that evaluated the associations of various baseline factors with the presence of nuclear cataracts, cortical cataracts, PSCs, and APCs.

**Table 2:** Odds ratio (95% CI) on multivariate analysis for different cataract subtypes (nuclear, cortical, PSC, and APC) in Korean.

	Nuclear (n = 1783)		Cortical (n = 807)		PSC (n = 56)		APC (n = 104)	
	MODEL1†	MODEL2‡	MODEL1	MODEL2	MODEL1	MODEL2	MODEL1	MODEL2
	OR(95% CI)	OR(95% CI)	OR(95% CI)	OR(95% CI)	OR(95% CI)	OR(95% CI)	OR(95% CI)	OR(95% CI)
**Age**								
40–49	1	1	1	1	1	1	1	1
50–59	4.19(3.12–5.63)*	4.19(3.12–5.63)*	2.57(1.84–3.60)*	2.57(1.84–3.60)*	1.61(0.45–5.77)	1.61(0.45–5.76)	4.94(2.12–11.5)*	4.98(2.13–11.62)*
60–69	10.01(7.18–13.96)*	10.02(7.19–13.97)*	4.75(3.29–6.86)*	4.76(3.29–6.87)*	1.96(0.57–6.74)	1.95(0.58–6.59)	3.11(1.25–7.72)*	3.12(1.26–7.73)*
70+	8.82(6.28–12.38)*	8.85(6.31–12.43)*	5.10(3.49–7.44)*	5.11(3.51–7.46)*	4.36(1.28–14.78)*	4.30(1.32–14.02)*	2.98(1.23–7.24)*	3.06(1.25–7.51)*
**Sex**								
Male	0.92(0.81–1.04)	1.07(0.94–1.22)	0.94(0.79–1.11)	1.05(0.89–1.25)	0.82(0.44–1.52)	0.9(0.51–1.60)	1.17(0.77–1.76)	1.27(0.83–1.92)
**Smoke**								
Never	1	1	1	1	1	1	1	1
Current smoker	0.92(0.77–1.08)	1.14(0.95–1.38)	0.76(0.62–0.94)	0.86(0.68–1.09)	0.42(0.14–1.24)	0.46(0.14–1.49)	0.89(0.47–1.71)	0.89(0.45–1.77)
**Education**								
Middle school or less	2.41(2.01–2.88)*	1.24(1.01–1.53)*	2.63(1.99–3.48)*	1.75(1.28–2.40)*	1.43(0.63–3.26)	0.81(0.41–1.59)	1.59(0.89–2.86)	1.19(0.54–2.63)
**Weight change** Φ								
Gain>10 kg	0.72(0.34–1.54)	0.95(0.43–2.11)	0.5(0.07–3.72)	0.61(0.08–4.79)	.	.	.	.
Gain≤10 kg	0.64(0.52–0.79)*	0.91(0.74–1.13)	0.81(0.6–1.11)	1.08(0.79–1.47)	0.82(0.29–2.32)	1.01(0.34–2.98)	0.76(0.37–1.58)	0.93(0.43–2.03)
No change	1	1	1	1	1	1	1	1
Loss≤10 kg	1.19(1.00–1.42)*	1.08(0.90–1.30)	1.13(0.87–1.45)	1.04(0.80–1.35)	1.53(0.72–3.27)	1.44(0.69–2.99)	1.78(1.03–3.07)	1.68(0.98–2.89)
Loss >10 kg	0.58(0.30–1.12)	0.43(0.22–0.84)*	0.81(0.28–2.28)	0.67(0.22–2.01)	.	.	.	.
**Daily sunlight exposure**								
More than 6 hours	1.27(1.06–1.51)*	1.07(0.88–1.30)	1.63(1.20–2.20)*	1.48(1.10–2.00)*	1.05(0.50–2.21)	0.98(0.46–2.08)	2.04(1.05–4.00)*	1.86(0.92–3.76)
**Alcohol** §								
Mild to moderate drinker	0.66(0.57–0.76)*	1.09(0.93–1.28)	0.71(0.58–0.87)*	1.06(0.85–1.33)	1.17(0.56–2.43)	1.71(0.74–3.96)	0.57(0.39–0.85)*	0.67(0.42–1.05)
Heavy drinker	0.68(0.51–0.91)*	1.23(0.9–1.69)	0.79(0.58–1.07)*	1.26(0.89–1.76)	0.2(0.05–0.86)*	0.32(0.06–1.62)	0.73(0.35–1.49)*	0.78(0.34–1.78)
**Daily sleep hour**								
4 hours or less	2.52(1.76–3.62)*	1.37(0.94–1.99)	1.53(0.91–2.59)	0.94(0.55–1.60)	3.08(0.82–11.56)	2.06(0.55–7.73)	0.35(0.05–2.64)	0.25(0.03–1.89)
4–6 hours	1.62(1.38–1.91)*	1.26(1.06–1.50)*	1.24(0.99–1.55)	1.00(0.79–1.25)	1.81(0.81–4.01)	1.52(0.71–3.27)	1.10(0.63–1.93)*	0.96(0.55–1.68)
More than 6 hours	1	1	1	1	1	1	1	1
**Stress level**								
Excessive	1.00(0.75–1.33)	1.41(1.05–1.91)*	1.04(0.67–1.60)	1.35(0.85–2.13)	0.58(0.07–4.62)	0.70(0.09–5.37)	1.50(0.61–3.71)	1.85(0.72–4.71)
Much	0.70(0.57–0.86)	1.15(0.93–1.43)	0.78(0.59–1.02)	1.15(0.86–1.54)	1.00(0.41–2.43)	1.35(0.55–3.33)	0.59(0.29–1.21)	0.81(0.38–1.70)
Frequent	0.69(0.58–0.83)	1.14(0.94–1.38)	0.80(0.63–1.01)	1.17(0.91–1.51)	0.54(0.23–1.26)	0.73(0.33–1.61)	1.20(0.67–2.12)	1.60(0.86–2.96)
Rare to never	1	1	1	1	1	1	1	1
**BMI**								
≥25 kg/m^2^	1.01(0.88–1.67)	1.08(0.94–1.25)	0.921(0.76–1.11)	0.97(0.79–1.17)	0.94(0.46–1.93)	0.98(0.48–1.98)	1.47(0.97–2.24)	1.53(0.99–2.36)
**Waist circumference**								
≥90 cm in male, ≥80 cm in female	0.71(0.62–0.82)*	0.81(0.70–0.94)*	1.08(0.87–1.34)	1.25(1.00–1.55)*	0.95(0.49–1.84)	1.10(0.61–1.97)	0.84(0.53–1.35)	0.87(0.54–1.40)
**Estrogen use (women only)**								
Yes	0.76(0.59–0.99)*	0.70(0.54–0.91)*	0.79(0.54–1.14)	0.74(0.51–1.06)	0.94(0.23–3.91)	0.77(0.19–3.21)	1.59(0.62–4.04)	1.52(0.59–3.93)
**Steroid use**								
Yes	1.06(0.79–1.42)	0.96(0.71–1.28)	1.28(0.86–1.91)	1.19(0.78–1.80)	2.95(1.64–13.52)*	2.72(1.57–12.96)*	0.39(0.16–0.93)*	0.37(0.17–0.84)*
**NSAIDs use**								
Yes	0.96(0.60–1.54)	1.04(0.64–1.70)	0.84(0.43–1.64)	0.88(0.44–1.73)	0.72(0.08–6.73)	0.81(0.09–7.17)	0.59(0.19–1.88)	0.64(0.20–2.04)
**Hypertension**								
Yes	1.97(1.67–2.31)*	1.21(1.03–1.43)*	1.60(1.33–1.92)*	1.11(0.90–1.37)	1.24(0.63–2.41)	0.84(0.39–1.82)	1.64(1.02–2.62)*	1.30(0.8–2.12)
**Diabetes**								
Yes	1.99(1.60–2.48)*	1.47(1.15–1.87)*	1.29(0.99–1.66)	1.00(0.77–1.32)	3.16(1.47–6.78)*	2.60(1.19–5.68)*	0.89(0.46–1.74)	0.74(0.39–1.43)
**Vitamin use**								
Yes	0.99(0.83–1.19)	0.95(0.79–1.14)	1.10(0.81–1.49)	1.07(0.79–1.45)	1.36(0.36–5.18)	1.33(0.35–5.06)	0.58(0.33–1.00)	0.55(0.31–0.95)*

PSC = posterior subcapsular opacity; APC = anterior polar cataract; OR = odds ratio; CI = confidential interval; BMI = body mass index; NSAIDs = nonsteroidal anti-inflammatory drugs.

† Model1 = no adjustment.

‡ Model2 = adjusted for age and sex.

* Statistically significant.

Φ Weight change after the age of 20 years.

§ Classified as nondrinker, mild-to-moderate drinker (1.0–30.0 g alcohol/day), or heavy drinker (>30.0 g alcohol/day) after conversion of the average frequency and amount of alcoholic beverages consumed into the amount of pre-alcohol (in grams) consumed per day.

### Nuclear cataracts

The prevalence of definite nuclear cataract increased significantly with increasing age. The factors associated with increased likelihood of nuclear cataract were low educational level (middle school or less) (OR, 2.41; 95% CI, 2.01–2.88; p<.0001), mild weight loss less than 10 kg since 20 years of age (OR, 1.19; 95% CI, 1.00–1.42; p<.0001), sunlight exposure greater than 6 hours (OR, 1.27; 95% CI, 1.06–1.51; p = 0.0089), sleep duration 4–6 hours (OR, 1.62; 95% CI, 1.38–1.91; p<.0001), hypertension (OR, 1.97; 95% CI, 1.67–2.31; p<.0001), and diabetes (OR, 1.99; 95% CI, 1.60–2.48;p<.0001) after adjustment for age and gender. The factors associated with decreased likelihood of nuclear cataract were a waist circumference of ≥90 cm (men) or ≥80 cm (women) (OR, 0.71; 95% CI, 0.62–0.82; p<.0001) and estrogen use in women (OR, 0.76; 95% CI, 0.59–0.99; p = 0.0426).

### Cortical cataracts

Cortical cataracts were associated with increasing age. A higher risk for cortical cataract was associated with lower educational level (OR, 2.63; 95% CI, 1.99–3.48; p<.0001), sunlight exposure greater than 6 hours (OR, 1.63; 95% CI, 1.20–2.20; p = 0.0015), and waist circumference ≥90 cm (men) or ≥80 cm (women) was associated with higher risk (OR, 1.25; 95% CI, 1.00–1.55; p = 0.0439) after adjustment for age and gender.

### Posterior subcapsular cataracts

Higher risk was associated with increasing age but was only statistically significant for the ≥70 years age group (OR, 4.36; 95% CI, 1.28–14.78; p<.0001). An increased risk of PSC was also associated with diabetes (OR, 3.16; 95% CI, 1.47–6.78; p = 0.0031).

### Anterior polar cataracts

The highest odds ratios were for the 50–59 years age group (OR, 4.94 ; 95% CI, 2.12–11.5, reference category 40–49 years; p = 0.0008). Oral steroid use was associated with a lower risk for APC (OR, 0.37; 95% CI, 0.17–0.84; p = 0.0171).

## Discussion

Increasing age was a significant risk factor for nuclear cataract, cortical cataract, and PSC, which is consistent with previous reports [2], [7], [12], [15], . In contrast to the results for other types of cataracts, the prevalence of and odds ratio for APCs were highest in the 50–59-year-old age group, suggesting that these cataracts develop during early adulthood; therefore, age may not be a significant factor in the pathogenesis of APC. This population-based study showed an APC prevalence of 3.78% among all participants, which broke down to 0.4%, 1.9%, 1.2%, and 1.2% in the 40–49-, 50–59-, 60–69-, and ≥70-year-old age groups, respectively. As APC is more likely to develop in early adulthood, the patients themselves may more readily detect the visual impairment and may therefore be more likely to seek early cataract surgery than patients with age-related cataracts.

We previously reported in a hospital-based study that 6.02% of all cataracts were APCs and that 87% of APCs were in men [16]. The differences in prevalence between the studies may result from the participant selection. The previous, hospital-based study was a chart review of the patients who underwent cataract surgery, while the present study was a cross-sectional investigation of the normal population. Men with APC have a greater tendency to undergo early surgical intervention than women because men are more socially active in Korea. Other explanation may be possible that since there was Korean war from 1950 to 1953, Koreans (especially, mothers and their babies) might underwent unusual experiences including nutrition, chemical exposures, and psychiatric stress. Still, the gender differences could not be solved if they were exposed as the same environment.

Diabetes was associated with a significantly higher risk for nuclear cataract and PSC. In contrast to the results of the AREDS study [7], cortical cataract was not significantly associated with diabetes in the current study. The Blue Mountains study confirmed diabetes as a risk factor for nuclear cataract and implicated impaired fasting glucose as a possible risk factor for cortical cataract [22]. Our results support the latter study. We also found that hypertension was associated with an increased risk of nuclear cataract. The results for hypertension regarding the cataract prevalence are inconsistent. The Blue Mountain Eye Study [22] reported a relationship between blood pressure and having cataract surgery and the Los Angeles Latino Eye Study [21] showed that systolic blood pressure to be an independent risk factor for PSC. The varying effect of different antihypertensive medications may be a confounding factor in all of these observational investigations and further prospective studies would be needed.

Our results revealed an increased risk for PSCs but a reduced risk for APCs in systemic steroid users. There is a possibility of an inflammatory etiology of APC since systemic steroids are primarily used for inflammatory activities. While the association between hormonal replacement therapy (HRT) and cataract development is inconsistent [7], [23], this study showed a lower risk for nuclear cataracts in estrogen users and a non-significant tendency toward an increased risk for APCs. Estrogens have antioxidant properties, but the possible protective effect of estrogen may be more associated with endogenous estrogen. Exogenous estrogen in the form of hormone replacement therapy should not be regarded as a physiologic substitute and could have other effects.

The current study revealed that vitamin use significantly reduced the risk for APC, but not for other types of cataracts, after adjustment for age and gender. Most of the large prospective studies investigated the effect of Centrum [7], [24], which contains a broad spectrum of vitamins and minerals, while our results are based on a survey of general vitamin use [7]. Identification of the components of Centrum that contribute to its protective effect against nuclear cataracts remains an area of active investigation.

Our study has some potential limitations. The KNHANES data were not collected for ophthalmologic evaluation, and much of the information is based on self-reporting. The information on smoking history, sunlight exposure, stress level, alcohol consumption, and medication use may therefore be biased. Recall bias may also confound the reporting of weight change since the age of 20 years. And although this study used photographic standards according to LOCS II classification, the gradings of the lens opacities were not investigated. We used the cut-off values for defining cataracts and this may be a weak point evaluating associated factors of cataracts. However, the strength of this study is that there have been few studies in high income Asian countries which would be interesting to compare to low and middle income Asian countries where the prevalence of cataracts were shown to be high. Also, the anthropometric measurements and diabetes and hypertension diagnoses in the current study were made by trained examiners and physicians, while many of the previous studies based this information on self-reporting. Finally, as Koreans have relatively uniform genetic and environmental influences, including a single race, climate, and food culture, the results may be more consistent than those of other large, population-based studies. Moreover, the exceptionally high prevalence of anterior polar cataract (APC) in the Korean population warrants a national data study to advance scientific knowledge and identify possible racial differences. Further studies on immigrants to Korea may provide a clue to the environmental and genetic contributors to the pathogenesis of APC.
